# AAV Recombineering with Single Strand Oligonucleotides

**DOI:** 10.1371/journal.pone.0007705

**Published:** 2009-11-02

**Authors:** Matthew L. Hirsch, Francesca Storici, Chengwen Li, Vivian W. Choi, R. Jude Samulski

**Affiliations:** 1 UNC Gene Therapy Center, University of North Carolina at Chapel Hill, Chapel Hill, North Carolina, United States of America; 2 Department of Pharmacology, University of North Carolina at Chapel Hill, Chapel Hill, North Carolina, United States of America; 3 School of Biology, Georgia Institute of Technology, Atlanta, Georgia, United States of America; 4 Harvard Medical School, Cambridge, Massachusetts, United States of America; Virginia Tech, United States of America

## Abstract

Adeno-associated virus (AAV) transduction initiates a signaling cascade that culminates in a transient DNA damage response. During this time, host DNA repair proteins convert the linear single-strand AAV genomes to double-strand circular monomers and concatemers in processes stimulated by the AAV inverted terminal repeats (ITRs). As the orientation of AAV genome concatemerization appears unbiased, the likelihood of concatemerization in a desired orientation is low (less than 1 in 6). Using a novel recombineering method, Oligo-Assisted AAV Genome Recombination (OAGR), this work demonstrates the ability to direct concatemerization specifically to a desired orientation in human cells. This was achieved by a single-strand DNA oligonucleotide (oligo) displaying homology to distinct AAV genomes capable of forming an intermolecular bridge for recombination. This DNA repair process results in concatemers with genomic junctions corresponding to the sequence of oligo homology. Furthermore, OAGR was restricted to single-strand, not duplexed, AAV genomes suggestive of replication-dependent recombination. Consistent with this process, OAGR demonstrated oligo polarity biases in all tested configurations except when a portion of the oligo targeted the ITR. This approach, in addition to being useful for the elucidation of intermolecular homologous recombination, may find eventual relevance for AAV mediated large gene therapy.

## Introduction

Adeno-associated virus (AAV) is a single-strand (ss) DNA dependovirus of the family, Parvoviridae. The 4.7 kb genome consists of two genes, *rep* (replication) and *cap* (capsid), flanked by 145 nucleotide (nt) inverted terminal repeats [ITRs, 1]. AAV transduction results in both polarity genomes within the host nucleus where they form duplexed DNA, either by annealing or by extension of the free 3′ end of the ITR [2]. The generation of the duplexed genomes is perhaps coincident with their circularization, preferably as monomers but also as concatemers [3]–[5]. The circularization events are stimulated by the ITRs, and the created double-strand episomes are stable and capable of long-term gene expression.

The past 20 years has demonstrated the modulation and effective application of AAV for gene therapy in humans. This approach relies on replacing the viral genes with a therapeutic cassette such that only the ITRs remain [referred to as recombinant or rAAVs, 6]. Regarding *in vivo* DNA delivery for therapeutic applications, rAAV is perhaps currently the most desirable method as the virus is considered non-pathogenic, has a minimal tendency for chromosome integration, results in sustained gene expression and the capsid can be designed for targeted transduction [7]. However, a major limitation of rAAV is its small packaging capacity (≈4.7 kb), rendering it deficient for therapies requiring large promoters and or transgenes. To overcome this limitation, research has exploited host cellular machinery to concatemerize distinct genomes delivered by separate capsids, resulting in a larger DNA molecule. Two slightly different approaches have been used *in vitro* and *in vivo* termed overlapping vectors, and more successfully, *trans*-splicing AAV vectors [referred to herein as split vectors as splicing technically occurs in *cis*; 4], [5], [8]. In fact, the later approach has demonstrated therapeutic relevance in a mouse disease model [9]. However, as AAV split vector genome concatemerization is unbiased, the efficiency of functional transgene reconstruction is low. An additional rate-limiting step is the resultant ITR junction, which impedes transcription and splicing of the reconstructed transgene up to 50% [10]. Therefore, improvement on the split vector approach should address the ability to exclusively direct productive dimerization while eliminating the inhibitory ITR junction.

Several reports demonstrate that AAV genomes induce a DNA damage response in the host cell including; I) AAV infection induces G2/M arrest [11], II) the DNA repair proteins involved in non-homologous end joining (NHEJ) and homologous recombination, Ku86 and Rad52 respectively, directly bind AAV genomes [12], III) microinjection of ITRs induces ATM checkpoint activation similar to DNA double-strand breaks [DSBs; 13], IV) AAV2 DNA triggers a damage response similar to a stalled replication fork which depends on ATR and Chk1 signaling [14], [15], and V) DNA-PKcs and Artemis process the AAV ITRs [16]. Collectively, these results provide evidence for the two canonical DNA repair pathways, NHEJ and homologous recombination, in the circularization/concatemerization of AAV genomes.

The efficiency of homologous recombination is stimulated several orders of magnitude by a DNA DSB within the repair target [17]. Consistently, DNA inverted repeat sequences are processed to DSBs [18] and stimulate gene correction with a homologous substrate, such as a single-strand oligonucleotide [oligo; 18], [19]. In the context of AAV concatemerization, it is possible that homologous recombination is also stimulated following processing of the ITRs [16]. Therefore, we hypothesized that the orientation of AAV concatemerization could be directed using a single-strand oligo complementary to distinct genomes functioning in ITR repair.

This report describes a novel recombineering method: Oligo-Assisted AAV Genome Recombination (OAGR). In this approach, a DNA oligo with homology to distinct transduced AAV genomes directs concatemerization via homologous recombination. Oligos targeting different regions of the transduced genomes varied in their ability to stimulate intermolecular recombination, which in some cases approached the efficiency of single vector transduction. Interestingly, oligo polarity biases for OAGR were observed similar to other oligo-mediated DNA repair processes in bacteria, yeast and other human contexts [20]–[23]. Characterization of the recombination junctions suggests a process in which the oligo homology tethers distinct single-strand genomes and host enzymes generate a single larger molecule precisely at that position. OAGR, in addition to being useful for the elucidation of intermolecular homologous recombination in a human context, may find eventual relevance for AAV mediated large gene therapy.

## Results

**Figure 1:**
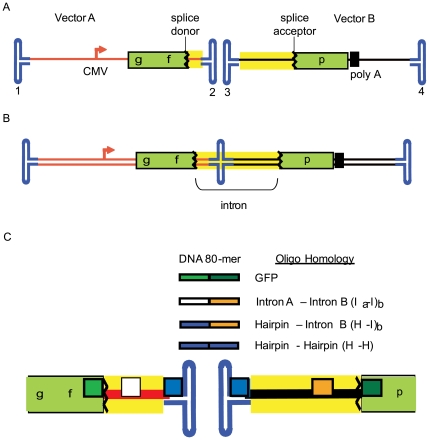
AAV Single-Strand Split *gfp* Vector System. A) Two AAV genomes are depicted, vectors A and B, that have the following genetic arrangement: i) vector A contains the CMV promoter upstream of a partial *gfp* coding sequence (cds) followed by a splicedonor site, and ii) vector B contains intron elements, the remainder of the *gfp* cds followed by a poly-adenylation signal. The inverted terminal repeats (ITRs) of the distinct genomes are labeled 1–4 and yellow highlights the intron region. B) The functional concatemer: a single DNA molecule for the ITR 2–3 linkage which results in functional GFP after splice removal of the intron. C) DNA 80-mers are depicted individually having 40 nt of homology to the color matched positions on vectors A and B. The crooked lines represent the splice donor and acceptor sequences and the intron is highlighted in yellow.

**Figure 2:**
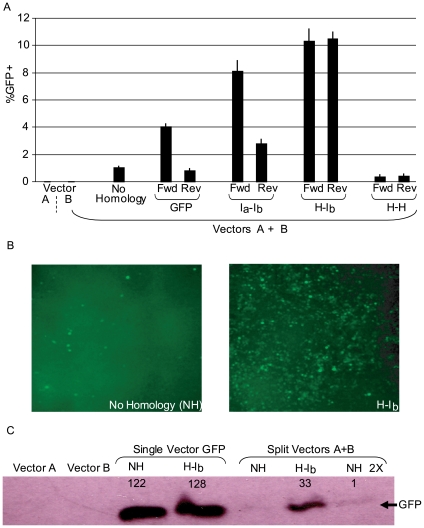
Oligonucleotides Direct AAV2 Concatemerization. A) Human embryonic kidney cells were co-infected with the split *gfp* vectors (A and B; Fig. 1) at a multiplicity of infection of 200 for each, vector A and B. Then, DNA oligonucleotides (80 nM) displaying 40 nucleotides of different sequence homology to vector A and B genomes were transfected in (see Fig. 1, non-homologous = NH). Three days post-treatment, GFP+ cells were quantitated by flow cytometry. An 80-mer without vector A and B homology was used as the non-homologous control (NH). The tested oligos are described (Fig. 1 and in the text) and opposite polarities were also investigated (Fwd = sense = non-template strand, Rev = anti-sense). Results are presented as %GFP + cells. B) Fluorescent microscopy of cells treated as in A) with a NH oligo or the H-I_b_ Fwd oligo used for transfection. C) Cells were treated as described in A). Additionally a single vector with the identical split gfp reporter as the split vectors (a CMV promoter upstream of the *gfp* coding sequence interrupted by the *hcg* intron) was investigated (single vector Gfp). Cells treated as in A) with the split vectors, or the single vector, followed by oligo transfection were harvested 3 days post-treatment. Western blotting was then performed to quantitate Gfp protein (NH 2X = twice the total protein loaded compared to all other lanes). Using densitometry, the relative levels of Gfp abundance was determined and the acquired values are also depicted.

To test the hypothesis that DNA oligos homologous to separate AAV genomes enhance functional concatemerization we initially employed ssAAV2 split *gfp* vectors (Fig. 1*A*). These consist of two distinct genomes, each having ITRs, with the respective features (termed vectors A and B throughout): A. a CMV promoter followed by the first 381 nt of *gfp* coding sequence at which point a canonical splice donor site was added and B. the human chorionic gonadotropin (*hcg*) intron and the remaining *gfp* coding sequence followed by a poly-adenylation sequence (Fig. 1*A*). Transduction by either vector alone does not result in functional GFP (Fig. 2*A*). We chose to evaluate this effect in human embryonic kidney cells (293s) due to the high efficiency of AAV2 transduction and DNA oligo transfection using PEI. Results from other gene targeting experiments demonstrated that DNA 80-mers served efficiently for chromosomal and episomal repair (Fig. S1; data not shown, DNS). Therefore, we exploited the function of 80-mers for the majority of experiments. As negative controls, several oligos without split vector genome homology were evaluated and similar results were obtained, the value of which is defined as natural concatemerization (Fig. 2*A*, no homology; DNS).

The initial oligo tested contained 40 nt of 3′ terminal *gfp* coding sequence homology to vector A and 40 nt of 5′ *gfp* coding sequence homology on vector B (Fig. 1*C*, GFP). Co-transduction of the ssAAV2 split *gfp* system, at a multiplicity of infection (MOI) of 200 for each vector, followed by transfection of the forward orientation (sense = non-template strand = Fwd) of the GFP oligo resulted in a significant increase (3 to 4 fold) of GFP+ cells compared to the non-homologous control after 72 h (Fig. 2*A*). The anti-sense version (Rev) of the same oligo did not alter the amount of GFP+ cells significantly from the non-homologous control oligo (Fig. 2*A*).

### Oligo-Assisted Intermolecular AAV Genome Recombination Depends on Oligo Target Homology

Next, oligos with different AAV genome targets were evaluated; i) 40 nt of homology to unique single-strand intron sequences of each genome (Fig. 1*C*, intron A-intron B = I_a_-I_b_), ii) an oligo with 40 nt of homology to the duplexed AAV Rep binding element (RBE) present in all AAV ITRs and 40 nt of homology to single-strand intron sequence exclusively on vector B (Fig. 1*C*, hairpin-intron B = H-I_b_) and iii) an oligo with the described H homology as an inverted repeat capable of binding all ITRs (H-H, Fig. 1*C*; Table S1). Co-transduction by the split single-strand *gfp* vectors followed by the transfection of the Fwd versions of I_a_-I_b_ or H-I_b_ resulted in an 8-fold or 10-fold increase in the amount of GFP+ cells, respectively, over the non-homologous control after 72 hours (Fig. 2). A decrease in GFP+ cells, below the non-homologous control, was observed after transfection of the H-H oligo in either orientation (Fig. 2*A*). Similar to the Gfp Rev oligo result, the I_a_-I_b_ Rev oligo did not perform as well as the Fwd version with only a modest enhancement of GFP+ cells (Fig. 2*A*). Interestingly, this was not the case for the H-I_b_ Rev oligo which increased the amount of GFP+ cells to the same extent as the equivalent Fwd version (10-fold).

It should be noted that the OAGR effect was found independent of the oligo manufacturer and comparable with standard desalting and PAGE purification methods (DNS). Additionally, split *gfp* AAV2 particles purified from cesium chloride gradients and different chromatographies were all equally subject to OAGR (DNS). Furthermore, OAGR effects were independent of the AAV capsid serotype (Fig. S2).

The effect of the H-I_b_ oligo on *gfp* reconstruction after ssAAV2 split *gfp* co-transduction was compared to the transduction efficiency of a single AAV2 vector containing the exact genetic elements of the split system, CMV-*gf*-*hcg* intron-*p*-polyA, on a single-strand genome. It was first noted that the H-I_b_ Fwd or Rev oligos did not alter the GFP+ transduction profile, or GFP abundance, of the single intact particle (Fig. 2*C*; Fig. S3). Then, the enhancement of the H-I_b_ Fwd oligo on split *gfp* vector co-transduction was compared to the efficiency of the intact *gfp* single vector in the presence of the same oligo. Following transduction (or co-transduction) with an equivalent number of viral genomes, there was an approximate 2-fold increase in GFP+ cells for the single intact *gfp* vector compared to cells treated with single-strand split *gfp* vectors and the H-I_b_ as measured by flow cytometry (Fig. S3). Quantitation of the GFP directly by Western analysis demonstrates that OAGR increases the efficiency of split vector gene reconstruction greater than 60-fold (Fig. 2C). This OAGR effect resulted in 28% of the GFP produced by the single intact vector while natural concatemerization produced only 0.4% (Fig. 2C).

**Figure 3:**
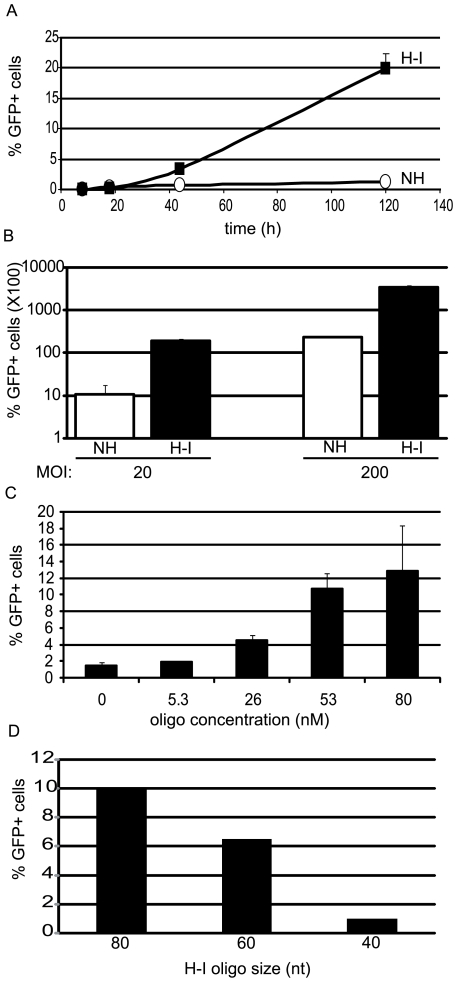
Characterization of Oligo-Assisted AAV Genome Concatemerization. A) Human embryonic kidney (HEK) cells were co-infected by AAV2 single strand split *gfp* vectors at a multiplicity of infection (MOI) of 200 for each vector. Then, either H-I_b_ Fwd or the non-homologous (NH) oligos were used for transfection (80 nM). GFP positive cells were quantitated by flow cytometry at the indicated times. B) HEK cells were co-transduced with the indicated MOI of each split *gfp* vector and subsequently transfected with the H-I_b_ Fwd oligo or a NH control (80 nM). Three days later the number of GFP+ cells was determined by flow cytometry. C) HEK cells were co-transduced with the AAV2 split *gfp* vectors at a MOI of 200 and the indicated concentrations of H-I_b_ Fwd was used for transfection. GFP+ cells were determined on day 3. The presented results were not normalized to the efficiencies of co-infection and oligo transfection. D) Oligo size analysis for stimulation of OAGR. Cells were co-infected with the split vector system as above and then either the H-I_b_ Fwd oligo or derivatives of that sequence were then used for transfection. %GFP positive cells were determined on day 3 by flow cytomtery. nt = nucleotide.

The time course of the H-I_b_ Fwd oligo effect on split *gfp* vector co-transduction was compared to a non-homologous control oligo. In this experiment, the cells were maintained for continuous replication. An increase in GFP+ cells was noted after 42 h for cells treated with the H-I_b_ oligo. Thereafter, the number of GFP+ cells increased with time in a linear fashion to over 20-fold greater than the control at day 5 (Fig. 3*A*). Southern blot analysis of intracellular oligo persistence demonstrated the greatest abundance at 24 h and stability out to day 5 under the PEI transfection conditions used herein (Fig. S4).

### Oligo-Assisted Intermolecular AAV Genome Recombination is Dependent on MOI

Next, the effect of H-I_b_ Fwd oligo was characterized at increasing MOIs of the AAV2 split *gfp* vectors. Three days after co-transduction/transfection, GFP+ cells were quantitated. At an MOI of 20, the H-I_b_ oligo increased GFP+ cells over 20-fold (Fig. 3*B*). This value was not statistically different from the use of 10-fold more particles and a non-homologous oligo (Fig. 3*B*). When a capsid less efficient for 293 cell transduction (AAV8) was used at an MOI of 20, functional reconstruction was only observed in the presence of the H-I_b_ oligo, while no GFP+ cells were noted for natural concatemerization (Fig. S3). These results highlight the inefficiency of AAV split vectors as well as emphasize the ability of OAGR to allow a 10-fold decrease in particle administration while maintaining the same level of gene reconstruction.

### Oligo-Assisted Intermolecular AAV Genome Recombination is Dependent on Oligo Abundance and Size

Initially, a high oligo concentration of 80 nM was chosen to emphasize any stimulatory effect (Fig. 2, Fig. 3*A, B*). We also transfected decreasing concentrations of the H-I_b_ Fwd oligo (or a non-homologous control) and measured GFP+ cells 72 h post-co-transduction (MOI 200). In this context, the number of GFP+ cells at increasing concentrations of the non-homologous oligo did not change and is statistically equivalent to the 0 nM concentration (Fig. 3*C*, DNS). A dose-dependent response to the H-I_b_ Fwd oligo was apparent with 26 nM being the first tested concentration at which an effect was significant (Fig. 3*C*). No significant difference in GFP+ cells was noted for concentrations 53 nM and 80 nM (Fig. 3*C*).

To determine whether smaller oligos could direct recombination of split vector genomes, DNA 60-mers and 40-mers based on the H-I_b_ Fwd oligo sequence, were also investigated. Decreasing the size of the oligo by 20 nt maintained a 6-fold OAGR effect while the 40-mer (20 nt arms of homology) was not significantly different from natural concatemerization (Fig. 3*D*). This observation is consistent with results from oligo-mediated DSB repair in yeast cells [19].

### Sequence Confirmation of Oligo-Directed Concatemerization

**Figure 4:**
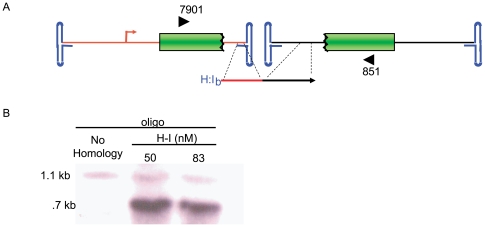
Junction Characterization of Functional Concatemers. A) This cartoon depicts our AAV split *gfp* vector system and the position of primers (block arrows) used for functional concatemer amplification. B. Evaluation of amplification products by southern blotting (*gfp* probe) showed a unique band formed only from DNA isolated for H-I treated cells. This band was directly cloned and sequenced.

If oligo directed concatemerization resulted from homologous recombination, then repair of distinct AAV genomes would create a junction corresponding to the sequence of the oligo. To investigate this, we performed PCR analysis of the functional concatemer junctions using DNA from co-transduced cells treated with the H-I_b_ Fwd oligo or the non-homologous control at different concentrations. PCR primers were designed and used to amplify recovered DNA external of any *gfp* functional junction (Fig. 4*A*; Materials and Methods). DNA harvested on day 3 from cells treated with the single-strand split *gfp* vectors and the H-I_b_ Fwd oligo (80 nM) resulted in a unique PCR product corresponding to the predicted size of the concatemer junction if recombination were to occur at H-I_b_ homology, (an approximate 400 bp deletion compared to the natural concatemer; Fig. 4). This product was not observed in cells receiving the non-homologous oligo and was subsequently cloned and sequenced. Sequencing of 20 clones demonstrated that the OAGR concatemer junction corresponded to the sequence of the oligo, effectively making a deletion that includes the ITR junction.

### Oligo-Assisted Self-Complementary AAV Recombination

**Figure 5:**
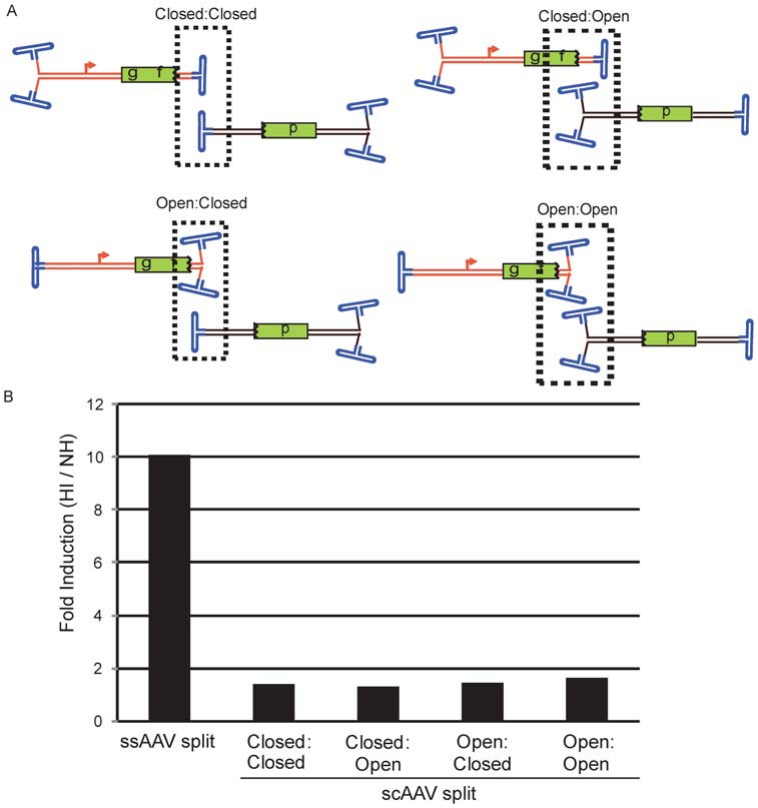
Oligo-Assisted AAV Genome Concatemerization of Self-Complementary Split *gfp* Vectors. A) Four split *gfp* systems are depicted for self-complimentary (sc) AAV2 genomes. The genomes differ in the orientation of their respective cassettes (see text for details) in relation to the open or closed ends of the molecule. A broken box encases the type of junction necessary for functional GFP. B) The pairs of split *gfp* vectors depicted in A were used for co-infection of human embryonic kidney cells at a multiplicity of infection (MOI) of 200 for each particle type. Then, either H-I_b_ Fwd or a non-homologous (NH) oligo was used for transfection. Three days later, GFP+ cells were determined by flow cytometry. The results are presented as fold-induction which is the number of GFP+ cells given the H-I_b_ oligo divided by the number of GFP+ cells given the NH oligo.

To determine if OAGR prefers a single-strand or a duplexed template, a self-complementary (sc) AAV2 split *gfp* system [5] was employed in our oligo assay (Fig. 5). In the sc context, a small deletion on only one of the ITRs results in transduced genomes that likely have a hinge (closed end = C) at one end of the complementarity and free, or open (O), ends on the other side (Fig. 5*A*). Thus, AAV genome dimerization resulting in functional GFP was investigated in all contexts (vector A-vector B; C-C, C-O, O-C, O-O; Fig. 4*A*). It is important to note that oligos tested on the single-strand system are also homologous to the scAAV2 genomes and that individual vector transduction does not result in GFP synthesis [5]. As performed above, 293 cells were co-transduced by these scAAV split vector combinations at an MOI of 200 for each vector, followed by transfection of an oligo and determination of GFP+ cells on day 3. Consistent with a previous report, concatemerization appeared most efficient with the C-C scAAV2 split *gfp* vector orientation and least efficient arranged in the O-O configuration (Fig. S5, 5). However, in contrast to the H-I_b_ Fwd OAGR effect on the single-strand vectors (Fig. 2*A*) only a minor to no effect was observed on the different self-complementary orientations (Fig. 5*B*). As the effect of the H-I_b_ Fwd oligo was more distinguishable at low MOIs, we also investigated the C-C vector combination under these conditions, however, no effect greater than 2-fold was observed (DNS). Additionally, no significant effect was observed with the GFP oligo (Fig. 1*C*) which targets a portion of the *gfp* coding sequence on the distinct genomes (DNS).

### Oligo-Assisted Intramolecular Genome Recombination

**Figure 6:**
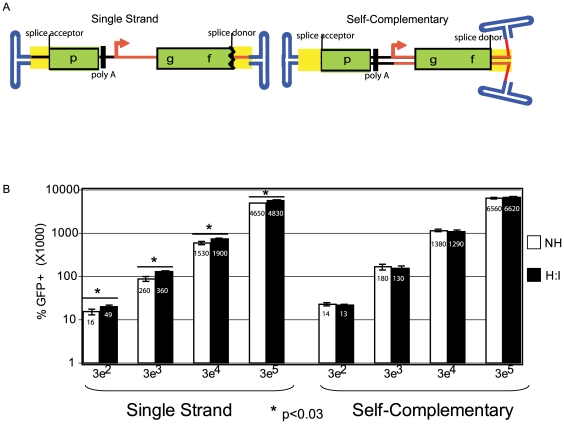
Oligo-Assisted AAV Genome Recombination of Circularization Dependent AAV. A) The cartoon depicts single vector split *gfp* systems that are capable of GFP production after either intra- or inter-molecular dimerization. The relevant system features are depicted; the arrow represents the CMV promoter followed by the 5′ portion of the *gfp* coding sequence (cds) and an splice donor sequence. On the far left side is the *hcg* intron and the remaining *gfp* cds followed by a poly-adenylation sequence. The entire intron is highlighted in yellow. Both the single-strand (ss) and self-complementary (sc) versions are shown. B) AAV2 capsids packaged with either genome depicted in A were used to separately infect human embryonic kidney cells at the indicated viral genomes per cell. Then, either the H-I_b_ Fwd or a non-homologous (NH) oligo was used for transfection. Three days later, GFP+ cells were determined by flow cytometry. In all cases the single-strand construct demonstrated a significant (unpaired student t test) increase, yet less than 2-fold, with the H-I_b_ Fwd oligo compared to the NH control. No significant changes were note for the sc construct.

GFP synthesis after ssAAV split *gfp* co-transduction results from a functional dimerization of vector A and B genomes. In comparison, single-strand and self-complementary vectors were generated, (using the same genetic elements as both the single-strand and self-complementary split *gfp* genomes described herein) that result in GFP synthesis after intramolecular linkage [circularization dependent; 5]. The genetic organization of these molecules follows: ITR-*hcg* intron-downstream *gfp* coding sequence-polyA-CMV promoter-upstream *gfp* coding sequence-intron-ITR (Fig. 6*A*). At low MOIs, these vectors depend on genome circularization (monomerization) for GFP synthesis, which has been reported by several groups to be preferred, in comparison to concatemer formation, at least initially [3], [5], [24]. Consistently, it was also reported that at low MOIs the self-complementary circularization dependent vectors were similar to genomes containing an intact transcriptional *gfp* cassette [5]. To investigate whether the H-I_b_ Fwd oligo promotes intramolecular circularization, 293 cells were transduced with either a single-strand or self-complementary version of the circularization dependent split *gfp* vector at increasing MOIs. At all vector to cell ratios tested, transfection of the H-I_b_ Fwd oligo slightly (less than 2-fold), yet significantly (p<0.03), increased the number of GFP+ cells compared to values determined with the control oligo for the single-strand vector (Fig. 6*B*). In contrast, no significant effect of the H-I_b_ oligo was noted for the self-complementary version (Fig. 6*B*).

## Discussion

In most dividing cells, AAV DNA initiates a signaling cascade that culminates in a transient G2/M cell cycle arrest. Following infection, there is pan-nuclear activation of γ-H2AX and host proteins involved in DNA repair process the linear single-strand viral DNA to double-strand circular monomers and concatemers. The results described herein exploit this aspect of the AAV life cycle to demonstrate the first example of directed concatemerization, specifically and exclusively, of distinctly transduced AAV genomes. This intracellular genome engineering technique relies on the ability of an oligo to tether DNA molecules and induce recombination at the site of homology. A dependence on a single-strand DNA substrate is suggestive of replication-dependent recombination and consistent with the observed oligo polarity biases in bacteria [25] and yeast [22]. In addition to serving as a unique example of facilitated recombination, the successful translation of OAGR to *in vivo* settings may allow efficient large gene delivery by distinct rAAV split vectors.

Using ssAAV split *gfp* vector co-infection followed by the transfection of oligos having complementarity to both split genomes, dramatic effects on GFP abundance/function were observed. These effects exhibited a broad range of variation dependent upon the region of the AAV genome targeted by the oligo (Fig. 2). The choice of the most efficient oligo target homology is not completely understood. However, analysis of additional oligos were not consistent with the obvious theories that decreasing the distance from the ITR (Fig. S6) or increasing the oligo-target annealing temperature result in an increased OAGR effect (Fig. S7). Surprisingly, the most effective oligo, H-I_b_, contains a 5′ 40 nt region capable of annealing to all ITRs and the 3′ 40 nt provide direction specificity by targeting an unique intron region found on vector B. If the 5′ region is shifted upstream of “H” homology to target a unique region of vector A, while maintaining the exact vector B homology (“I_b_”) there is slight reduction in the OAGR effect (Fig. 2*A*). Perhaps the observed decrease represents a separate additional effect specific to targeting the ITR (i.e. an effect on second-strand synthesis). Alternatively, the tetrad repeat of the ITRs (GACT) may represent a recombination hot-spot sequence as it is the origin of replication and involved in host chromosome integration as well [6].

A polarity bias for OAGR was noted for the GFP and I_a_-I_b_ oligo sequences but not for the H- I_b_ oligo that proved most effective (Fig. 2*A*). Since the vector B homology was constant between I_a_-I_b_ and H-I_b_, this difference is attributed to the 5′ portion of the oligo. At this position, the I_a_ homology targets a single-strand region unique to the vector A intron while the “H” sequence targets a region common to all ITRs. Since the H target is paired, it is capable of binding both genome polarities while the vector A “I_a_” homology would be restricted to its complement. Common to all oligos that demonstrated a polarity bias is the fact they target a region that undergoes second-strand synthesis, whereas the oligo that functions equally in both polarities (H-I_b_) targets the duplexed region which is not synthesized in the generation of no end AAV genomes. This brings to mind an explanation for the polarity bias initially derived from single-strand oligo chromosome repair in *Escherichia coli* [20]. In that case, it was observed that the oligo strand bias correlated with direction of replication through the recombination site [20]. This result suggested that lagging strand synthesis would allow greater access to its template strand and consistently, oligos complementary to that strand out performed the opposite orientations. In the case of the AAV bi-polarity single-strand genomes, both oligo polarities have equal opportunity for genome binding and continuous replication is initiated from the free 3′ end and therefore, imposes no obvious bias. As the OAGR event is postulated similar to replication-dependent recombination, an alternative model in which the ITRs are biased for their ability to initiate second-strand synthesis is proposed. In fact, polarity biases for AAV replication and genome packaging have been suggested previously [26], [27]. If replication-dependent recombination is responsible for the bias observed in this work, it argues that the oligo acts before second-strand synthesis which is supported by no significant OAGR effect on scAAV, or for an ITR containing split *gfp* plasmid system (Fig. 5, Fig. S5 and DNS).

In contrast to the results using the single-strand split *gfp* vectors, the scAAV genomes, and double-strand plasmids (DNS), were not responsive to homologous oligos, nor was there an effect for the intramolecular recombination of the scAAV circularization dependent vector (Fig. 6). This OAGR requirement for ssAAV genomes likely reflects the ability of the oligo to access its target. For example, the self-complementary molecule is essentially a 2 kb hairpin precluding oligo annealing. Another theory for the lack of the OAGR effect on the scAAV genomes is that OAGR is coupled to second-strand synthesis. As replication forks are “inherently recombinogenic” one can envision the annealed oligo blocking second-strand synthesis in a manner that would stimulate recombination of tethered, yet distinct, genomes [25]. Interestingly, only a minor OAGR effect of the H- I_b_ oligo was observed for the single-strand circularization dependent vectors which, at higher MOIs, could also undergo dimerization to generate GFP. This result supports that intramolecular genome linkage is very efficient, as has been reported [5], [24]. In this scenario, monomerization readily occurs and the modest increase observed with the homologous oligo is perhaps a result of intermolecular dimerization by OAGR, which is evident when monomer formation does not result in reporter activity (split vectors; Fig. 2*A*). This result supports a role for oligo tethering of distinct DNA molecules as opposed to a stimulation of intramolecular ITR processing or a simple model in which the oligo opens the ITRs. The absence of any oligo effect with the scAAV circularization dependent vector is consistent to the results obtained with the scAAV split *gfp* co-infection, and could be explained in the same manner (see above). Given that the activity of the single-strand and self-complementary circularization dependent vectors are somewhat similar without a homologous oligo (Fig. 6), the explanation for genome tethering by the oligo is further supported, especially when the 145 nt ITRs are likely the major local attraction. This also suggests that processing of single-strand and self-complementary genomes for concatemerization are similar. Additionally, we speculate that natural ITR-mediated circularization/concatemerization is primarily reliant on NHEJ which is more efficient than OAGR's reliance on homologous recombination, especially for an intramolecular linkage event.

**Figure 7:**
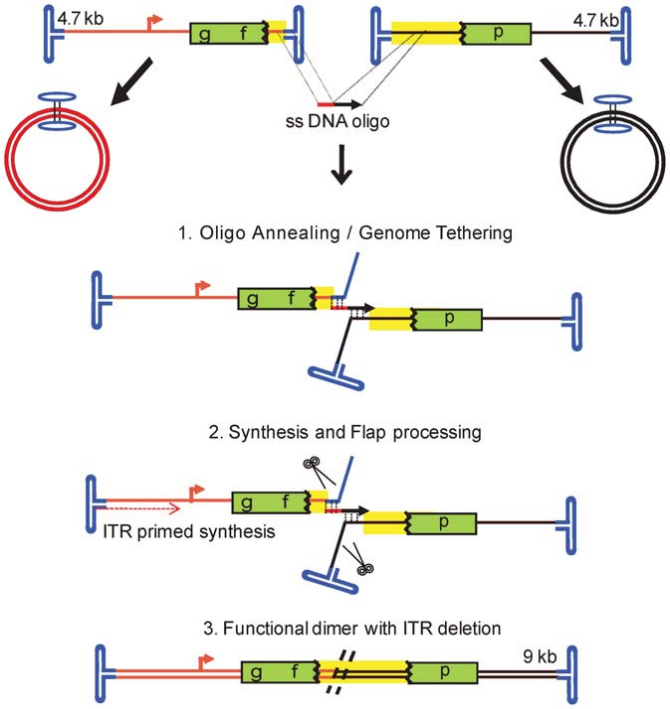
A Model for Oligo-Assisted AAV Genome Recombination. Following the capsid release of the 4.7 kb AAV genomes, presumably in the nucleus, oligos with homology to each vector anneal to their single-strand (ss) target, displacing the remaining terminal ends of the genome. This annealing event promotes the desired genome dimer orientation and viral genome replication using the inverted terminal repeats for initiation. Extension of the 3′ oligo end is also a possibility. Exploitation of host repair enzymes for intermolecular recombination follows resulting in a double strand product with the vector junction sequence corresponding to that of the oligo. In the case of the H-I_b_ oligo this recombination event results in an approximate 400 bp deletion of the ITR junction. The recombined dimer is near double the length of the independent genomes. Although it was not conclusively demonstrated, this model favors physical incorporation of the transfected oligo into the oligo-directed concatemer (described in discussion). Also depicted are perhaps the naturally favored products initially following transduction, circular monomers, which do not produce GFP.

The collective results support the “annealing–integration” model as the mechanism of OAGR (Fig. 7), similar to the “rounds of strand annealing” mechanism of DSB repair driven by single-strand DNA oligos in yeast cells [18] and in line with the model proposed for single-strand oligo recombineering in *E. coli* [20]. Following the capsid release of AAV genomes, oligos with homology to each single-strand vector genome anneal to their target, displacing the remaining terminal ends of the genome. This annealing event promotes the desired genome dimer orientation and exploits host replication machinery and DNA repair enzymes for intermolecular recombination with the resultant junction sequence corresponding to the sequence of the oligo. Evidence from additional modified oligos suggests that the annealed oligo physically incorporates into the newly formed dimer molecule, as demonstrated in both prokaryotes and human cell lines, acting as a split with or without oligo-primed synthesis [28], [29]. However, the exact mechanism is still under investigation.

## Materials and Methods

### Maintenance of cells

Human embryonic kidney (HEK) 293 cells were grown in Dulbecco's modified Eagle's medium supplemented with 10% heat-inactivated fetal bovine serum and 1X Penn/Strep (DMEM+, Sigma). Cells were grown at 37°C in a 5% CO_2_ humidified incubator.

### rAAV vector construction, production and purification

The self-complementary (sc) split *gfp* vectors and the sc *gfp* circularization dependent (cd) vectors have been previously reported [5]. The construction of the single-strand (ss) AAV-cd and ss split gene vectors was the same as the scAAV-cd and sc split-gene vectors, with the exceptions that different pieces of stuffer fragments were used, and that the expression cassettes were cloned into the pSSV9 [6] vector to generate conventional ssAAV vectors. Lambda Phage *Dra* I 3332 bp fragment was used as a stuffer for the upstream (vector A; Fig. 1*A*) ss split vector ssAAV-Gf using *Dra* I-5′-2 5′CTA CGC GTC ATC GCC AAT AAA AGT GGC G as a forward primer and *Dra* I-3′ 5′ GCA CGC GTA AAA GGC AGG TGG GC as a reverse primer. Lambda *Msc* I 3605 bp fragment was the stuffer for the downstream (vector B; Fig. 1*A*) ss split vector ssAAV-Gfp using *Msc* I-5′ 5′CTA CGC GTC CAV GCA GCT TGC AG as a forward primer and *Msc* I-3′ 5′GTA CGC GTC CAG CAT GAT ACG TCC CG as a reverse primer. The lambda *Mlu* I 956 bp fragment described above was used as a stuffer for ssAAV-*gfp* for an intact *gfp* cassette clone [5].

rAAV was generated using the triple transfection method with AAV serotype 2 capsid and purified by discontinuous iodixanol gradient separation and heparin chromatography [5], [30], [31]. Additional viral preparations, including rAAV8, were purified on a CsCl gradient, followed by peak fraction collection and dialysis against phosphate-buffered saline (PBS)[31]. The number of genome containing particles was determined by Southern dot blot hybridization while rAAV2 infectious units (MOI) were determined by infectious center assays in C12 cells [32].

### rAAV transduction

50,000 293 cells were seeded in a 24 well plate 24 h prior to transduction. The next day, the total medium volume was adjusted to 700 ul and virus particles were diluted in 50 ul of DMEM+ prior to well addition. A MOI of 200 I.U. of each split vector was used for transduction, unless otherwise noted. At the indicated time point (usually 72 h), cells were harvested by gentle aspiration and re-suspended in PBS for flow cytometric analysis. In the experiments described herein, the co-transduction efficiency was not explicitly determined, thus the experimental results were not corrected for co-transduction and likely under represent the actual values. All experiments were performed in triplicate on at least 3 different days and the results are presented as averages.

### Oligo transfection

The sequence of the oligos used in these studies listed in Table S1. The indicated concentration of oligo (80 nM if not indicated) was transfected 10 min post co-transduction using linear polyethylenimine (PEI) transfection reagent prepared in house. In these experiments, the oligo was diluted in DMEM without supplements, vortexed in the presence of PEI, and the mixture was added to the well 10 min later where it remained unless otherwise noted. Under these conditions, transfection of an equivalent amount of GFP expressing plasmid results in 70% GFP+ cells. However, the % GFP+ values presented in this work are not corrected for the efficiency of oligo transfection and thus, under represent the actual efficiency of the approach.

### Analysis of GFP+ cells

GFP+ cells were visualized by fluorescent microscopy and quantitated by flow cytometry. A minimum of 10,000 similar sized/shaped single cells were counted, for each replicate, at the indicated times at an event per second rate of 1500–2000. The GFP+ gate was designed such that untreated cells did not give a single GFP+ event beyond a million counted cells. It was also positioned away from false, or transitioning, GFP+ cells to obtain no false positives. Visualization of pooled GFP+ cells after FACs sorting confirmed the GFP+ phenotype (DNS).

### Concatemer junction sequence verification

GFP cells were pooled by FACs analysis and total DNA was recovered using a DNA-EZ kit (Qiagen). PCR reaction conditions were performed by standard procedures using pfu turbo polymerase (stratagene). The sequence of primers used to initially amplify a functional *gfp* concatemer follow (5′ to 3′ orientation): i) early *gfp* sequence on the upstreamsplit genome, CGGCGAGGGC GAGGGCGATG CCACC and ii) late *gfp* coding sequence (cds) on the downstream split genome, GACCGCCGCC GGGATCACTC TCGGC. Nested primers amplified the gel isolated product for cloning and subsequent sequencing: EcoRI-fwd CGCGAATTCGCTTCAGCCGC TACCCCGACC ACATG, Xba-rev gcga tctaga AGGTAGTGGTT GTCGGGCAGC AGC. In validation of this primer set, capsid purified A and B vector genomes (at different concentrations) served as templates in a PCR reaction where different amounts of the H-I_b_ oligo were spiked in the reaction (DNS). In these instances, a product corresponding to the oligo-directed concatemer junction could not be generated (DNS).

## Supporting Information

Table S1 Table of oligonucleotide sequences used in this study
(0.03 MB DOC)

Figure S1 Repair Oligonucleotide Size Influences Gene Correction. The chromosomal homologous recombination GFP reporter system of M. Porteus (Porteus et al. Mol Cell Biol. 2003 May; 23 (10):3558-65) was used to evaluate gene correction by different sized homologous oligonucleotides in the presence and absence of a Sce-induced double-strand break (DSB). In this system the stop codons in all reading frames and the Sce binding site is inserted into the gfp coding sequence. This defective reporter does not produce GFP until the insertion is releived. The numbers refer to the size of the oligonucleotides used for repair having equal sized gfp homologous arms to each side of the disrupting insertion. GFP+ cells indicates chromosomal reporter repair and fluorescence was determined 48 h post-transfection (oligonucleotide with or without a Sce expression plasmid) by flow cytometry. NH = non-homologous control oligonucleotide.
(0.97 MB EPS)

Figure S2 Oligo-Assisted Genome Recombination using AAV serotype 8 capsids. Human embryonic kidney cells were co-infected with the split gfp vectors (A and B; Fig. 1) packaged in serotype capsid 8 at a multiplicity of infection of 50,000 for each, vectors A and B. Then, the H-I DNA oligonucleotide (80 nM) displaying 40 nucleotides of sequence homology to vector A and B genomes was used for transfection (see Fig. 1, non-homologous = NH). Three days post-treatment, GFP+ cells were quantitated by flow cytometry. An 80-mer without vector A and B homology was used as the negative control (NH).
(1.45 MB EPS)

Figure S3 Comparison of Oligo-Assisted Genome Recombination on Intact and Split AAV vectors. Human embryonic kidney cells were infected with either a single vector having a split gfp gene system (see text) or the identical split gfp gene system encoded on separate vectors (A and B; Fig. 1) at a multiplicity of infection of 200. Then, the H-I DNA oligonucleotide (80 nM) displaying 40 nucleotides of different sequence homology to vector A and B genomes, or non-contiguous homology to the single intact gfp vector (see text), was used for transfection (see Fig. 1, non-homologous = NH). Three days post-treatment, GFP+ cells were quantitated by flow cytometry. An 80-mer without vector A and B homology was used as the negative control (NH).
(0.95 MB EPS)

Figure S4 Oligonucleotide persistence in 293 cells. The NH or H-I oligonucleotides (described in text) were transfected to 293 cells using PEI. At the indicated timepoints total DNA was recovered and separated on an alkaline gel. Then, the DNA was transferred to a membrane and probed with the H-I oligonucleotide labeled with P-32. The positive control is 10 ng of the H-I oligonucleotide loaded on the gel directly.
(2.34 MB EPS)

Figure S5 Oligo-Assisted AAV Recombination of Self-Complementary AAV Split Vectors. Gene reconstruction from distinct transduced self-complementary AAV genomes was monitored in the presence of an oligonucleotide (H:Ib) having homology to both split vectors (see text for split vector description and Figure 5 for a cartoon depicting the vectors). Given the nature of self-complementary genomes 4 orientations exist in which gene reconstruction was evaluated (x-axis; see text and Figure 5). Gene reconstruction results in GFP+ cells which were evaluated on day 3 post-transduction and transfection by flow cytometry.
(1.10 MB EPS)

Figure S6 Evaluation of Oligonucleotide Target Homology for Oligo-Assisted AAV Genome Recombination. The sequence evaluated for oligo-assisted AAV genome recombination of the single-strand AAV split vectors (see text) is shown (the dotted lines project the evaluated region). A single oligonucleotide consists of 40 nucleotides (nt) of homology to Vector A (numbers) and 40 nt of homology to Vector B (letters). The single-strand gfp split vector system (see text and Fig. 1) was used for 293 cell transduction followed by transfection of the depicted oligos (the gfp oligo is described in the text). Three days later gene reconstruction was evaluated by flow cytometry. RBE = the AAV Rep binding element, TR = terminal repeat, cds = coding sequence.
(1.25 MB EPS)

Figure S7 Analysis of melting temperature in relation to Oligo-Assisted AAV Genome Recombination. Human embryonic kidney cells were co-infected with the single strand split gfp vectors packaged in serotype capsid 2 at a multiplicity of infection of 200 for vector A and B. Then, the oligonucleotides depicted displaying 40 nucleotides of sequence homology to vector A (H, H2) and B (I, I2) genomes were used for transfection (see Fig. 1). In these cases, the homology of the oligo to the AAV vector system was shifted slightly to significantly increase oligonucleotide/vectors annealing. Three days post-treatment, GFP+ cells were quantitated by flow cytometry. An 80-mer without vector A and B homology was used as the negative control (NH). Tm = melting temperature.
(1.28 MB EPS)
