# Supplementary material for: AAV Recombineering with Single Strand Oligonucleotides
Source: PLoS One. 2009 Nov 2;4(11):e7705. doi: 10.1371/journal.pone.0007705 (PMC2765622; doi:10.1371/journal.pone.0007705)
Supplement: Table S1 — Table of oligonucleotide sequences used in this study (0.03 MB DOC) [file pone.0007705.s001.doc]

**Supplementary Table 1. OAGR Oligonucleotides**

Lys Fwd
GTTAGTTCACTGGGTTTATCCATATGCCAAATTGAGGGACCCAAATGTTATTTCAACTATCAATGTTATGAGCTTAGCCG

Lys Rev
CGGCTAAGCTCATAACATTGATAGTTGAAATAACATTTGGGTCCCTCAATTTGGCATATGGATAAACCCAGTGAACTAAC

Gfp cds Fwd (80 nt)

GTTCGAGGGCGACACCCTGGTGAACCGCATCGAGCTGAAGGGCATCGACTTCAAGGAGGACGGCAACATCCTGGGGCACA

Gfp cds Rev (80 nt)

TGTGCCCCAGGATGTTGCCGTCCTCCTTGAAGTCGATGCCCTTCAGCTCGATGCGGTTCACCAGGGTGTCGCCCTCGAAC

Intron-Intron Fwd (80 nt)

GATCCTAGAGCATGGCTACGTAGATAAGTAGCATGGCGGGATGCCAGGAAGGGTCTCTGGGTCTTTGTGGGTGGTGTACC

Intron-Intron Rev (80 nt)

GGTACACCACCCACAAAGACCCAGAGACCCTTCCTGGCATCCCGCCATGCTACTTATCTACGTAGCCATGCTCTAGGATC

Hairpin-Intron Fwd (79 nt)

GGCCACTCCCTCTCTGCGCGCTCGCTCGCTCACTGAGGCATGCCAGGAAGGGTCTCTGGGTCTTTGTGGGTGGTGTACC

Hairpin-Intron Rev (79 nt)

GGTACACCACCCACAAAGACCCAGAGACCCTTCCTGGCATGCCTCAGTGAGCGAGCGAGCGCGCAGAGAGGGAGTGGCC

Hairpin Fwd (79 nt)

GGCCACTCCCTCTCTGCGCGCTCGCTCGCTCACTGAGGCGCCTCAGTGAGCGAGCGAGCGCGCAGAGAGGGAGTGGCCA

Hairpin Rev (79 nt)

TGGCCACTCCCTCTCTGCGCGCTCGCTCGCTCACTGAGGCGCCTCAGTGAGCGAGCGAGCGCGCAGAGAGGGAGTGGCC

The sequence of oligonucleotides (oligos) used in this study are listed below their respective name. All sequences are shown in the 5’ to 3’ orientation and Fwd means the forward sequence while Rev actually means the antiparallel (complementary) sequence with respect for the equivalent Fwd sequence. Letter in black correspond to split *gfp* vector A homology while red letters refer to that of vector B. The size of the oligos is also given (nt = nucleotides, cds = coding sequence).
